# Gene Network Reconstruction by Integration of Prior Biological Knowledge

**DOI:** 10.1534/g3.115.018127

**Published:** 2015-03-30

**Authors:** Yupeng Li, Scott A. Jackson

**Affiliations:** * Center for Applied Genetic Technologies, University of Georgia, Athens, Georgia; † Institute of Plant Breeding, Genetics and Genomics, University of Georgia, Athens, Georgia; ‡ Department of Statistics, University of Georgia, Athens, Georgia 30602

**Keywords:** gene network, graphical model, prior knowledge, systems biology and gene expression

## Abstract

With the development of high-throughput genomic technologies, large, genome-wide datasets have been collected, and the integration of these datasets should provide large-scale, multidimensional, and insightful views of biological systems. We developed a method for gene association network construction based on gene expression data that integrate a variety of biological resources. Assuming gene expression data are from a multivariate Gaussian distribution, a graphical lasso (glasso) algorithm is able to estimate the sparse inverse covariance matrix by a lasso (L_1_) penalty. The inverse covariance matrix can be seen as direct correlation between gene pairs in the gene association network. In our work, instead of using a single penalty, different penalty values were applied for gene pairs based on *a priori* knowledge as to whether the two genes should be connected. The *a priori* information can be calculated or retrieved from other biological data, *e.g.*, Gene Ontology similarity, protein-protein interaction, gene regulatory network. By incorporating prior knowledge, the weighted graphical lasso (wglasso) outperforms the original glasso both on simulations and on data from Arabidopsis. Simulation studies show that even when some prior knowledge is not correct, the overall quality of the wglasso network was still greater than when not incorporating that information, *e.g.*, glasso.

A key challenge for biology is to understand the complex molecular interactions of genes in a living cell (Barabasi and Oltvai 2004). Analysis of gene networks provides a global view of these interactions and can provide biologists with a better understanding of complex biological systems (Friedman 2004; Karlebach and Shamir 2008). Also, by the use of gene networks, the guilt-by-association paradigm can be applied to infer the biological function of unknown genes (Wolfe *et al.* 2005).

Gene expression data, which is relatively easy to generate or to collect from databases, has been used to infer gene networks. A variety of gene network reconstruction methods based on gene expression data have been developed, *e.g.*, regression, mutual information, correlation Bayesian network, meta predicators, and others (Marbach *et al.* 2012). Here, we focus on the Gaussian graphical model (Dempster 1972). In this approach, gene expression is assumed to follow a multivariate Gaussian distribution *N(μ*, Σ*)*, and Gaussian Markov random fields have been used to infer the structure of networks from gene expression data (Liu and Ihler 2011). A gene association network can be seen as an undirected graph *G = (V*, *E)*, where *V = {v_p_}* is the vertex set representing genes and *E = {e_ij_}* is the edge set representing association relations between pairs of genes. In an unweighted network, if *e_ij_* = 0, genes *i* and *j* are conditionally independent given all other genes. The Hammersley–Clifford theorem implies that zeros in the inverse covariance matrix of a multivariate Gaussian distribution indicate absent edges in the corresponding graphical model (Besag 1974; Lauritzen 1996). Therefore, the problem of estimating the gene association network based on gene expression data can be transferred to estimating *Σ^−1^* or selecting nonzero entries in *Σ^−1^*. Many studies, however, have used Pearson correlation coefficients between pairs of genes to infer network structure; Pearson correlation coefficients correspond to the covariance matrix Σ and cannot infer the true structure.

An accurate inference of biological network using Gaussian graphical model is challenging for two main reasons. The first is that most genome-scale datasets are highly dimensional. Given *p* genes, there are possible *p(p − 1)/2* edges, but gene expression data often have a limited number of samples. When the gene or locus number is much higher than the sample size in a dataset, a traditional maximum-likelihood estimate for covariance variance is often not appropriate (Schafer and Strimmer 2005; Uhler 2012). Alternative methods have been developed for highly dimensional datasets (Meinshausen and Buhlmann 2006; Friedman *et al.* 2008; Yuan 2010; Cai *et al.* 2011; Ravikumar *et al.* 2011), most of which have used Lasso, a shrinkage and selection method using *L_1_* regularization, a popular approach to deal with highly dimensional datasets (Tibshirani 1996; Hastie *et al.* 2009). These methods have been shown to be able to asymptotically and consistently estimate the set of non-zero elements of *Σ^−1^*.

The second major difficulty is the lack of efficient methods to integrate multiple levels of biological data to enhance model accuracy. Increasing amounts of biological data have been collected, especially with the development of high-throughput technologies, *e.g.*, microarrays and next-generation sequencing, which provide an unprecedented opportunity to explore biological systems. We now have access to genomic, epigenomic, transcriptomic, proteomic, metabolomic, and phenomic data, and careful analysis and integration of these genome-scale datasets should provide large-scale, multidimensional, and insightful views into biological systems (Joyce and Palsson 2006; Hawkins *et al.* 2010). Also, we have a variety of resources that serve as indicators of the functional association of any two genes, *e.g.*, Kyoto Encyclopedia of Genes and Genomes pathway, Gene Ontology (GO) similarity, protein−protein interaction, co-occurrence in literature, gene network generated from other methods, and association of orthologous genes. Integrating potentially reliable information from other sources should increase the accuracy of network reconstruction (Imoto *et al.* 2004; Mostafavi *et al.* 2008; Christley *et al.* 2009; Wang *et al.* 2013; Chen *et al.* 2014). Here, we present a statistical algorithm based on the Gaussian graphical model for gene association network reconstruction using gene expression data, which is able to solve these two challenges to allow a more thorough understanding of complex biological systems.

## Materials and Methods

Let *Θ = Σ^−1^* and S be the empirical covariance matrix, then by applying *L_1_* penalty to the original log-likelihood for estimating *Θ*, the problem of estimating *Θ* becomes finding the *Θ* which maximize the formula

$$\log\left(\det\left(\Theta \right)\right)-tr\left(S\Theta \right)-\rho {\left\|\Theta \right\|}_{1}\;,$$

where *tr* is the trace, *i.e.*, the sum of the elements on the matrix diagonal, and ${\left\|\Theta \right\|}_{1}$ is the *L_1_* norm, *i.e.*, the sum of the absolute values of the elements of *Σ^−1^*, and *ρ* is the penalty parameter. When *ρ = 0*, it is the normal maximum likelihood. When *ρ > 0*, some elements in *Θ* will be shrunk to zero. The sparsity of the estimated graph increases when *ρ* increases. For a fixed *ρ*, the graphical lasso (glasso) algorithm can be used to quickly solve the equation using a block-coordinate method (Friedman *et al.* 2008). The optimal *ρ* can be chosen empirically or tuned by cross-validation, Bayesian information criterion (BIC), or other methods (Friedman *et al.* 2008; Foygel and Drton 2010; Liu *et al.* 2010).

The Lasso regression can be interpreted as a Bayesian regression with a Laplace prior distribution, *Lp(0*, *ρ)*. In Bayesian statistics, prior information can be integrated into the model by changing the parameter in the prior distribution, and the posterior distribution should better approximate the true distribution of the data. Therefore, for the glasso algorithm, instead of using a single penalty parameter, it is reasonable to specify different amounts of penalties for different elements in *Θ* based on *a priori* information as to whether two genes are associated, or not. A smaller penalty can be given if *a priori* information indicates that they are linked. Then, the log-likelihood becomes

$$\log\left(\det\left(\Theta \right)\right)-tr\left(S\Theta \right)-\rho {\left\|P*\Theta \right\|}_{1}\;,$$

where *P* is the prior information matrix and * indicates component-wise multiplication, and P∈[0,1]. Larger values for an element in *P* generate larger penalty values and represent weaker association of two genes based on *prior*i information. We name this updated version of glasso as weighted graphical lasso (wglasso), which is not only stable for high-dimensional datasets by utilization of Lasso but also more accurate than the original glasso through the integration of prior information.

The prior matrix can be obtained in numerous ways. For example, the GO semantic similarity between genes can be calculated using tools GOSemSim (Yu *et al.* 2010) or GOssTo (Yu *et al.* 2010), Then, the inverse of similarities can be implemented in the prior matrix, as a high similarity means a low penalty in our model. Other types of networks, *e.g.*, protein-protein interaction and gene regulatory networks, can be directly transferred to a prior matrix after inverse transformation of the weights of the network. Some databases estimate the functional association between genes using data mining and text mining and can be valuable and reliable resources for generating a prior matrix, *e.g.*, STING (Von Mering *et al.* 2005) and AraNet (Lee *et al.* 2010).

Although previous efforts to use lasso for incorporating *a priori* knowledge have been made, none is identical to our proposed method. For example, most of the previously described algorithms partitioned the gene network reconstruction into a number of linear regression problems (Anjum *et al.* 2009; Charbonnier *et al.* 2010; Wang *et al.* 2013); however, it has been shown this type of algorithms is less accurate than glasso (Friedman *et al.* 2008).

## Results and Discussions

### Simulation studies demonstrate that wglasso is more reliable than glasso

**Figure 1:**
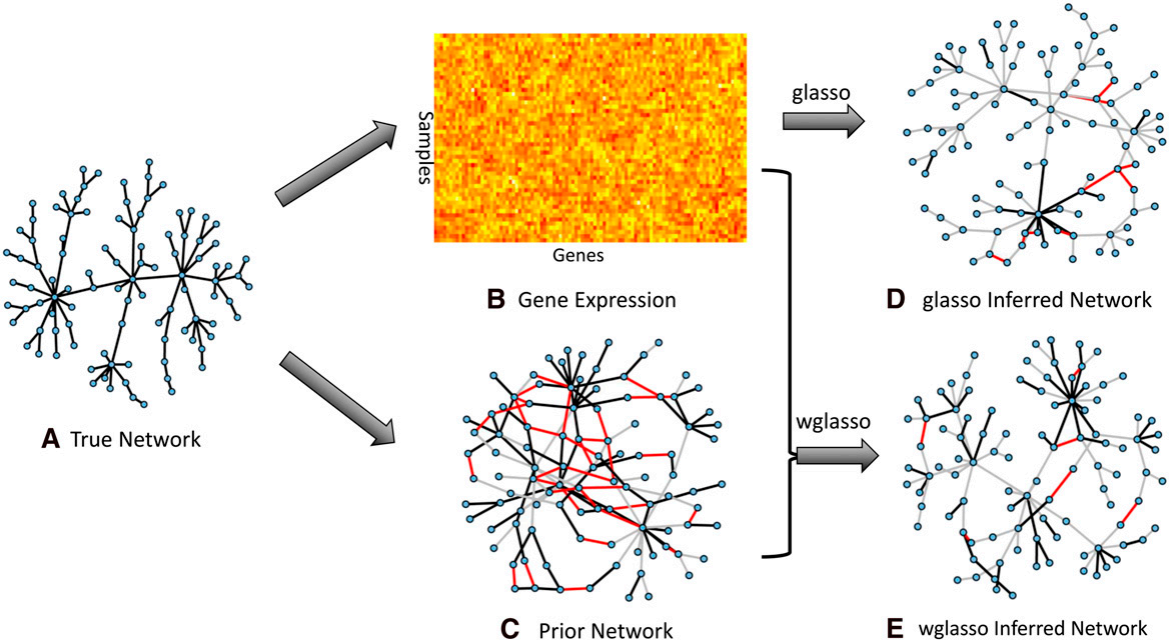
Demonstration of a single simulation using glasso and wglasso. (A) The true network with scale-free property. (B) Heatmap of gene expression data. The X-axis represents genes and the Y-axis represents samples. (C) The prior network, representing prior information of genes’ associations with precision ratio 0.7. Black edges are correct association information; edges that exist in prior information but not in the true network are in red, and gray edges are missed associations in the prior information. (D) The estimated network using glasso. The edge colors have the same meaning as prior network. (E) The estimated network using wglasso. The edge colors have the same meaning as prior network.

**Figure 2:**
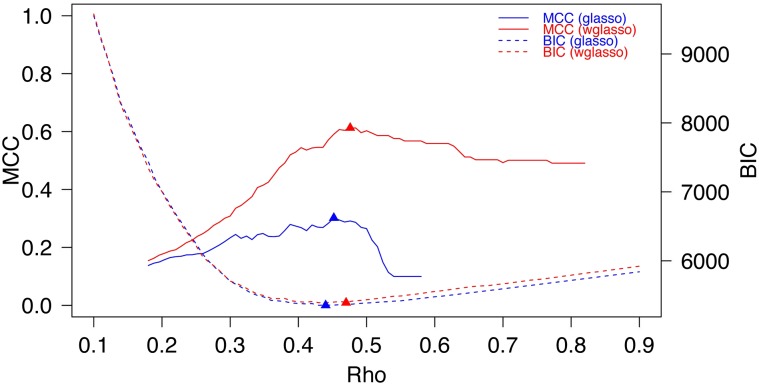
Parameter selection process of glasso and wglasso Matthews correlation coefficient (MCC; solid lines) and Bayesian information criterion (BIC; dashed lines) values of the estimated networks under different penalty parameters (Rho) using glasso (blue lines) and wglasso (red lines). Triangles are points that maximize MCC values and minimize BIC values under the corresponding penalty parameters, considered to be optimal penalty parameters.

Because most biological networks are scale-free (Barabasi and Oltvai 2004), a scale-free network with *p* genes and gene expression data with *n* samples based on the network topology are generated using the “huge” package in R (Zhao *et al.* 2012). A prior network with precision ratio *x*, *x ∈ [0,1]* also was generated. A prior network is a weighted network, in which each edge corresponds to the prior information that two genes are functionally associated and the edge weight indicates the strength of the information. Precision ratio = *x* with *x > 0* means p*100 percent of prior edges are correct, and the remaining (1 − p)*100 edges are incorrect. The edge number of the prior network is set to the same number of true edges. The edge weights of the prior network were randomly generated from the uniform distribution, *U(0,1)*. A special case is precision ratio = 0, which indicates no prior information and the prior network will not contain any edges. In this case, wglasso is equivalent to glasso. Once the empirical covariance matrix *S* is calculated from the expression data and the prior information matrix *P* retrieved from the prior network, we estimate the true network using glasso and wglasso. In order to find the optimal penalty parameter, *ρ*, wglasso networks were estimated under a sequence of *ρ* values from 0 to 1 with 0.01 intervals. Then we selected the network most similar to the true network. The Matthews correlation coefficient (MCC) was used to measure the similarity between the estimated and true networks (Matthews 1975), and was calculated as follows based on 2 × 2 contingency table,

$$MCC=\frac{TP\times TN-FP\times FN}{\sqrt{\left(TP+FP)(TP+FN)(TN+FP)(TN+FN\right)}},$$

where TP, TN, FP, and FN are the number of true positives, true negatives, false positives, and false negatives, respectively. A penalty parameter that maximized MCC (maxMCC) was considered optimal. A single simulation with 100 genes, 50 samples and a 0.7 precision ratio is shown in Figure 1 and Figure 2.

**Figure 3:**
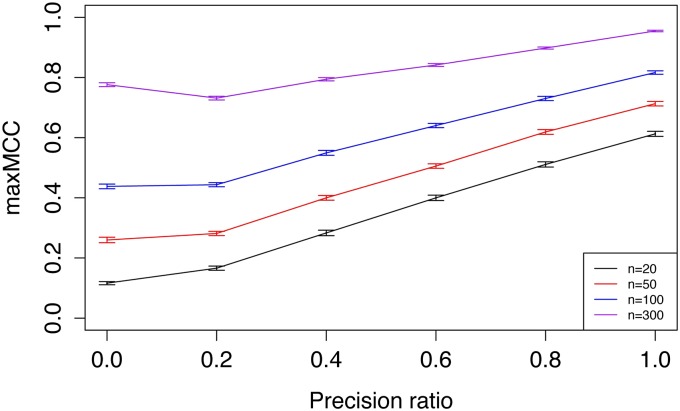
Simulation to compare glasso and wglasso. The simulation was repeated 100 times for each combination of sample sizes (n) and precision ratio of the prior information. Gene number = 100. In each simulation, the maximum Matthews correlation coefficient (maxMCC) of estimated networks from different penalty parameters is recorded. The Y-axis shows the mean of maxMCC from the 100 simulations. The error bars are 95% confidence intervals.

Additional simulations were performed with a variety of sample sizes and precision ratios in order to systematically evaluate wglasso. The simulation was repeated 100 times for each combination of sample size and precision ratio. The results show that reconstructed networks with *a priori* information had a significantly greater maxMCC than without *a priori* information, indicating superior performance of wglasso (Figure 3). In most cases, wglasso outperformed glasso, even when most of the prior knowledge was incorrect. If the sample size was high and the precision ratio low, *e.g.*, sample size = 300 and precision ratio = 0.2, it is possible that an excess of incorrect prior information would be harmful. However, in real situations, the sample size is often much lower than the gene number and misleading *a priori* information based on experimental studies is likely to be low. Moreover, highly accurate prior knowledge results in more accurately reconstructed networks. In practice, it would be possible to integrate multiple resources to increase the reliability of prior information.

### Tuning the penalty parameter using BIC

In real situations, the true network is unknown; thus, maxMCC cannot be used to select the optimal penalty parameter. Instead, one can use cross-validation, BIC, extended BIC (Foygel and Drton 2010), and a stability approach for regularization selection (Liu *et al.* 2010). Cross-validation, stability approach for regularization selection, and BIC conduct many subsampling or permutations and thus are computationally intensive. Extended BIC tends to select very sparse networks, and often no edges are found. For a scale-free network, another method is to test whether the log transformed degree distribution has a linear relationship (Langfelder and Horvath 2008). The R-squared values from linear regressions generally increase as the network gets sparser, and the optimal fit is selected at the point where the increase trend slows down. The selection is usually done visually, so the method is not practical for simulations. Also, the optimal parameter varies by dataset and individual interpretation.

**Figure 4:**
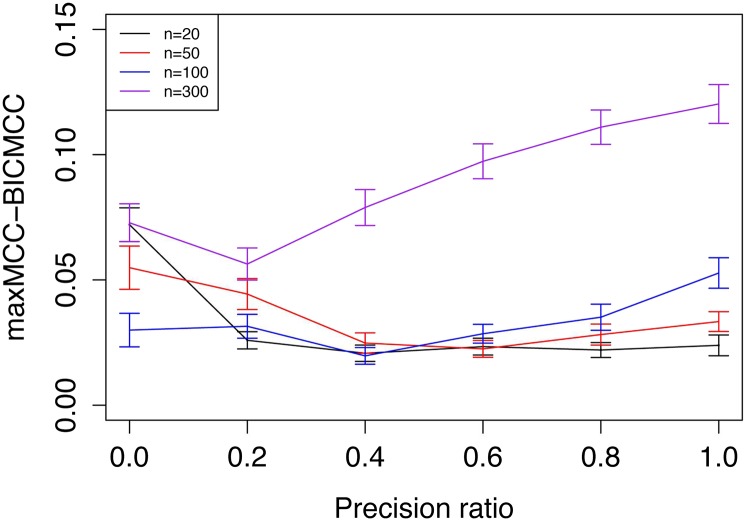
Selection of the optimal penalty parameter based on minimum Bayesian information criterion (BIC) simulation was repeated 100 times for each combination of sample sizes (n) and precision ratio of the prior information. Gene number = 100. For each simulation, the maximum Matthews correlation coefficient (MCC) value (maxMCC) of estimated networks from different penalty parameters is shown. The MCC value of the estimated network based on minimum BIC is also recorded, called BICMCC. The difference between two values (maxMCC − BICMCC) is used to evaluate two parameter selection methods. The Y-axis shows the mean of maxMCC − BICMCC from the 100 simulations. The error bars represent 95% confidence intervals.

BIC is one of the standard methods for choosing the regularization parameter, and works well in our scale-free network simulations (Figure 2 and Figure 4). The BIC for Gaussian graphical model takes the form

$$BIC=-2l_{n}\left(\text{Θ}\right)+\left|E\right|\log\left(n\right),$$

where |E| is the edge number, n the sample size, and $l_{n}\left(\text{Θ}\right)$ the log-likelihood function simplified from

$$l_{n}\left(\text{Θ}\right)=\frac{n}{2}\left[\log\left(\det\left(\Theta \right)\right)-tr\left(S\Theta \right)\right].$$

Simulation showed that the difference between the MCC of reconstructed networks based on BIC and the maxMCC was small, especially when sample size was relatively small (Figure 2 and Figure 4).

### Application to experimental data

We applied wglasso to gene expression data from the Eukaryotic species, *Arabidopsis thaliana*. Gene expression data of 795 genes related to isoprenoid pathways from 118 microarray experiments were collected (Wille *et al.* 2004). Prior information was obtained from AraNet, a probabilistic network of functional associations among 19,647 *Arabidopsis* genes (Lee *et al.* 2010). A total of 701 of 795 genes have functional associations in AraNet. The edge weight is the likelihood score, calculated from variety of resources that indicates a functional association between two genes. The likelihood scores range from 0 to 5, so they were rescaled to a range of 0.5 to 1 using the formula,

$$X\_new=0.5\times \frac{\max\left(X\right)-X}{\max\left(X\right)-\min\left(X\right)}+0.5.$$

High likelihood scores will become low penalty values after rescaling. Pairs of genes without prior information have zero values in the prior matrix. The optimal penalty parameter was selected based on BIC. The network reconstructed using wglasso has 16,759 edges, whereas the glasso network has 19,331 edges. The MCC values were calculated by comparing the reconstructed networks with an independent benchmark network, which is the same benchmark set that was used to test the prediction performance of AraNet (Lee *et al.* 2010). The MCC of the estimated network with wglasso was 0.184, higher than that with glasso, which was 0.033.

By incorporating prior knowledge, weighted graphical lasso (wglasso) outperforms glasso both on simulation studies and data from *Arabidopsis*. Simulation studies showed that even when some prior knowledge was incorrect, the overall quality of network from wglasso network was higher than that from glasso. Moreover, the more accurate the prior knowledge, the better the reconstructed network. This method increases gene network reconstruction accuracy and will allow researchers to better study networks in complex biological systems and their interaction with external factors, *e.g.*, the environment.
